# Perceptions of ICT Practitioners Regarding Software Privacy

**DOI:** 10.3390/e22040429

**Published:** 2020-04-10

**Authors:** Edna Dias Canedo, Angelica Toffano Seidel Calazans, Eloisa Toffano Seidel Masson, Pedro Henrique Teixeira Costa, Fernanda Lima

**Affiliations:** 1 Department of Computer Science, University of Brasília (UnB), P.O. Box 4466, Brasília-DF 70910-900, Brazil; PHTCOSTA@gmail.com (P.H.T.C.); ferlima@unb.br (F.L.); 2 University center—UniCEUB, Brasília-DF 70790-075, Brazil; angelica.toffano@gmail.com (A.T.S.C.); eloisa.masson@ceub.edu.br (E.T.S.M.)

**Keywords:** data privacy, ICT practitioners’ perception, general law of personal data protection, software development process, privacy requirements

## Abstract

During software development activities, it is important for Information and Communication Technology (ICT) practitioners to know and understand practices and guidelines regarding information privacy, as software requirements must comply with data privacy laws and members of development teams should know current legislation related to the protection of personal data. In order to gain a better understanding on how industry ICT practitioners perceive the practical relevance of software privacy and privacy requirements and how these professionals are implementing data privacy concepts, we conducted a survey with ICT practitioners from software development organizations to get an overview of how these professionals are implementing data privacy concepts during software design. We performed a systematic literature review to identify related works with software privacy and privacy requirements and what methodologies and techniques are used to specify them. In addition, we conducted a survey with ICT practitioners from different organizations. Findings revealed that ICT practitioners lack a comprehensive knowledge of software privacy and privacy requirements and the Brazilian General Data Protection Law *(Lei Geral de Proteção de Dados Pessoais, LGPD, in Portuguese)*, nor they are able to work with the laws and guidelines governing data privacy. Organizations are demanded to define an approach to contextualize ICT practitioners with the importance of knowledge of software privacy and privacy requirements, as well as to address them during software development, since LGPD must change the way teams work, as a number of features and controls regarding consent, documentation, and privacy accountability will be required.

## 1. Introduction

In Brazil, software development organizations, public or private, that process users’ personal data must comply with a large number of regulations and ensure that business and system requirements are legally compliant, namely, they implement the Brazilian General Data Protection Law *(Lei Geral de Proteção de Dados Pessoais, LGPD, in Portuguese)* in all their software systems.

Inappropriate use of data from users is subject to penalties if violated. However, knowledge of how to achieve data protection compliance is still quite limited from the point of view of software development teams. Thus, data privacy has become a significant concern to project managers and software development teams, mainly due to various incidents related to unauthorized data exploitation, misuse of personal information stored on social media sites, and disclosure of personal information to third parties without the consent of the data owners [1,2]. In addition, authors of different papers report the lack of understanding or knowledge in software privacy by the developers, and, in some cases, they do not know how to develop privacy-sensitive systems [3].

Data privacy violations can be prevented if privacy requirements are properly elicited during the early stages of software development, that is, in the specification phase of functional and non-functional requirements. Although much work has been developed proposing methodologies for privacy requirements’ elicitation [4,5,6,7,8,9,10,11,12], we found few works in the literature that have conducted empirical studies to describe how the software industry faces problems related to software development teams perceptions of system privacy [3,13,14], as well as what knowledge these professionals have, in order to perform correct implementations of these requirements along with the compliance with current legislation [13].

This paper aims to conduct a systematic literature review to identify the models and techniques used in the literature to implement software privacy and privacy requirements. Moreover, it aims to conduct qualitative research with Information and Communication Technology (ICT) practitioners in the software development industry to identify what is the perception of these professionals regarding software privacy, privacy requirements, and LGPD. In addition, to investigate what level of knowledge the development team members have concerning what the literature determines and classifies as software privacy and privacy requirements and how developers should implement these requirements and work through the software development process.

The main findings of this research were: (a) ICT practitioners’ perception regarding software privacy and privacy requirements corroborates with existing research [13], as software developers do not consider themselves fit to develop/implement this activity assuredly; (b) ICT practitioners’ knowledge of the LGPD, to be implemented in 2020, is not sufficient for the development of their activities in projects in which they work; (c) The organizational environment interferes with privacy practices; (d) ICT practitioners believe that changes proposed by LGPD will impact the activities of the requirements elicitation and development phases; (e) There are organizations in which ICT practitioners have not been informed about LGPD and its implementation or possible necessary changes in their current and future systems.

This article is organized as follows. Section 2 provides a background on concepts related to privacy and the LGPD, as well as related work. In Section 3, we present thoroughly the method adopted to conduct this work. In Section 4, we present the results of this study and the answers to the research questions that were defined, as well as discussions regarding the results. Threats to validity are presented in Section 5 and finally, in Section 6, the conclusions and future work are shown.

## 2. Background and Related Works

### 2.1. Systems’ Privacy

Privacy is not a new subject. In 1967, Westin [15] defined privacy as “people’s desire to freely choose under what circumstances and to what extent they will expose their attitude and behavior to others”. Over time, it became possible to identify that privacy “is a fluid and dynamic concept that has developed in parallel with technological and social changes” [16].

In the context of ICT, Dennedy et al. [17] define data privacy as a form of privacy derived from substantive privacy. According to Dennedy et al. [17], Substantive Privacy describes the individual’s right and ability to define and live his life in a self-determined manner. Thus, data privacy comprehends the data of the individual, created by him or others, and its use through observations, analysis, etc., by other individuals. Considering information systems, system privacy has been widely analyzed in recent years, especially with the advent of legal regulations and international standards [18,19,20].

Protecting the private data included in software systems is a complicated issue that encompasses several factors: legal regulations and restrictions, international standards, methodological support for developing systems in compliance with those regulations and restrictions, organizational environment, developer perceptions, and technologies [3,13,14]. It was possible to find some studies in the literature that highlight how privacy can be impaired or protected depending on the developed system design [21].

There are several proposals directed towards building an appropriate privacy system design, including the Privacy Engineer Manifesto, in which the authors [17] show recommendations of technologies and architectures to design privacy, models, and guidelines to include privacy in the initial stages of system requirements and systems design [22,23].

However, it is still unclear how effective these proposed models and guidelines are, and what are the possible limitations for implementing privacy in software engineering practice. Some authors identify the organizational climate (and its relationship to privacy) as a factor influencing the behavior of software developers in implementing privacy in the development of software products [3,13]. Organizations that promote their privacy, supervise, adopt educational and communication means to ensure that employees are conscious of and adhere to the organizational privacy policy, create an organizational climate of privacy awareness, enabling the implementation of privacy in their processes and products [3]. Therefore, the existence of organizational privacy policies is also considered an impact factor in this context.

Different authors identify developers’ knowledge of privacy—its values and beliefs—as an influence factor for privacy enforcement [13,14]. These authors further mention that developers’ knowledge of laws and regulations and organizational privacy policies plays an important role in implementing privacy more effectively [13,14]. Different works mention that developers’ knowledge and/or the use of existing privacy tools such as: encryption, user’s transparency, user’s control, turn off data collection, user’s access, automatic expiration date, data anonymization, decentralization, user’s deletion, and temporal data, among others, assist with the recognition and implementation of these tools and others in the context of system development [3,24].

Studying what are the impact factors in the implementation of system privacy is indispensable for organizations to define actions to optimize the software development process, which begins with eliciting functional, non-functional, and privacy requirements.

### 2.2. Privacy Requirements

Privacy has become one of the main concerns in software development, mostly due to incidents related to unauthorized exploitation of data, misuse of information stored on social media websites, internet data, disclosure of personal information to third parties without the consent of users [1]. Some participants in the software development process have no knowledge or understanding of privacy, and do not know how to develop privacy sensitive systems [3].

It is possible to prevent privacy violations if privacy requirements are correctly identified/elicited during the initial stages of software development, at the requirements’ specification stage. Privacy is the ability of an individual to control their information [6]. Thus, privacy becomes more relevant in the way users rely on software to achieve their regular activities [6]. Privacy specifications are the ones that comprehend the privacy purposes and their associated means for a system development [25]. Privacy goals may include: identification, authentication, authorization, data protection, anonymity (to preserve a user’s name), pseudonymity (identifier of a subject rather than the actual name of the subject), and unlinkability and unobservability (meaning that, within a system, an attacker cannot distinguish sufficiently whether or not items of interest are related) [6].

Users’ privacy requirements of a system may go beyond legal requirements. They must also be considered in software design, in order to obtain user acceptance. Engineering approaches to existing privacy requirements differ in their methods for obtaining privacy requirements and in their results [26]. There are three basic privacy principles: data minimization, enforcement, and transparency. These principles are requirements that must be specified during requirements’ elicitation and should be taken into account in the software development phase [26], as well as functional, non-functional, security, and privacy requirements.

### 2.3. Privacy and Design

Since the emergence of the Internet in the mid-1990s, the rapid development of information technologies has led to the use of increasingly invasive privacy technologies. The debate about the need for technological solutions that protect privacy first emerged in Europe in 1995, and the concept of Privacy by Design emerged at that time [27].

Privacy by Design (PbD) concept was devised to mitigate privacy threats since the early stages of the software development process [14]. This approach is based on seven fundamental principles that consider user privacy as a central aspect of organizational practices. They are [23]:

- Be proactive and preventive;
- Adopt privacy as a standard (defining specification purpose, collection limitation, data minimization, use limitation, retention, and disclosure);
- Incorporate privacy into design;
- Ensure full functionality (incorporation of privacy must be implemented without compromising functionality);
- Ensure security and protection throughout the whole data lifecycle;
- Give visibility and transparency (with accountability, openness and compliance);
- Respect user privacy.

Although the concept of PbD has several promises, researchers analyze it for being abstract and hard to achieve [28]. Despite the apparent simplicity of the idea of PbD, the big challenge lies in translating the meaning of privacy defined in rules and regulations—which were subsequently instituted—[18,19,20], into concrete guidelines for software developers. In addition, how will these developers implement this using their current development models, such as Agile Methods, Waterfall Model, Unified Process, and so on, and with what tools? Thus, the idea of designing privacy in ICT systems has been the subject of much debate in several organizations and domains. Moreover, in the literature, there are some works that aim to formulate privacy standards for ICT system design [17,20], but efforts to deal with privacy using technical means are still scattered and disconnected [29].

In an attempt to solve this problem, Privacy Engineering proposes an emerging research structure, interdisciplinary that concentrates on designing, implementing, adjusting, and evaluating theories, processes, techniques, and tools to address privacy issues in systems development systematically [29]. Privacy Engineering, according to Gurses and Álamo [29], comprises the following aspects:

- Privacy engineering methods that are approaches to systematically capture and address privacy issues during the development, management, and maintenance of information systems;
- Privacy engineering techniques that relate to procedures (a prescribed language or notation) for performing privacy engineering tasks or activities; and
- Privacy engineering tools (automated) to support privacy engineers during part of a privacy engineering process.

According to Gurses and Álamo [29], empirical studies are still needed to explore how privacy issues are (or are not) currently addressed in different engineering contexts. In their view, it is crucial to assess which methods, techniques, and tools are most appropriate in a given software privacy context complying with current laws and regulations.

### 2.4. Brazilian General Data Protection Law (LGPD)

On 14 August 2018, Brazil passed Law Number 13,709—Brazilian General Data Protection Law (LGPD) [18] which stipulates the protection of personal data and is expected to enter into force in 2020. Its nature is extensive in relation to the territorial and personal scope. Considering the territorial scope, the law applies to organizations in Brazil and also organizations that are not physically located in Brazil, however, provide goods and services or process personal data in Brazil.

With a focus on personal scope, the main reason for the Act is the processing of personal data of individuals (information related to the identified natural person, such as name, age, marital status, documents) performed by controllers and processors [18]. The Act also provides special protection for the processing of children’s personal data and sensitive personal data. Data related to the identified natural person, such as special data on racial or ethnic principle, political conviction, religious belief, association to a congregation or religious, philosophical or political affiliation, data associated with health or sex life, biometric, and genetic data [18]. Regarding individual rights, LGPD [30]:

- Allows individuals to request the deletion of their personal information unless an exception applies;
- Requires controllers to provide people with a detailed privacy notice, presenting information about the processing of their personal data;
- Gives data subjects the right to object to the processing of their personal data;
- Explicitly recognizes the principle of non-discrimination as a fundamental principle of data protection;
- Recognizes the right to data portability for data subjects. This concerns personal data determined as information regarding an identified or identifiable individual.

Considering the context of data privacy, several models were proposed with principles similar to LGPD, among them ISO/IEC 29100–Information technology–Security techniques–Privacy framework [20] and the General Data Protection Regulation (GDPR) [19,31]. GDPR entered into force in the European Union (EU) on 25 May 2018, through the Regulation EU 2016/679 [19].

According to DataGuidance by OneTrust [19], LGPD and GPDR have many similarities with a few disagreements regarding the processing of individuals’ personal data. Table 1 presents a macro comparison between the principles, rights, legal bases of the LGPD model [18], and GDPR [19,31]. LGPD provides 10 principles while GDPR 7. We decided to present all LGPD principles and complement them with individual rights and legal bases corresponding to GDPR. Table 1 demonstrates that, regardless of whether it is considered a legal principle or basis, privacy concerns are common to both models.

It must be highlighted that ISO/IEC 29100 [20] has 12 principles and most part of these principles are similar to GDPR [19,31] and LGPD principles [18], other principles are referred as “individual rights” or “legal bases”. For example, Consent and Choice is a principle in ISO/IEC 29100 [20]; however, it is considered a legal basis/individual right in LGPD and GDPR.

Regardless of the model adopted by the country or organization, several authors identify the need to study the views of information and communication technology (ICT) practitioners on privacy and the organization’s position on privacy, among other aspects [3,13].

## 3. Study Settings

In this section, we detail the surroundings of our study. Firstly, we state the goal of our research. Then, we present details about our research questions. Furthermore, we explain the procedures we took to conduct the study. Finally, we collect issues from the elicitation privacy requirement.

### 3.1. Research Goal

The main goal of this study is to create a broad comprehension about how ICT practitioners understand the guidelines of the LGPD and how they are being addressed by software development teams and by project managers in organizations. Furthermore, we conducted a research in the literature of the current scenario regarding the models and techniques of software privacy specifications and privacy requirements, in addition to the perception of privacy that ICT practitioners engaged in software development projects have.

### 3.2. Research Questions

We carried out a multi-method study to investigate the following research questions:

1. RQ.1. According to the literature, what are the methodologies and techniques used to conduct software privacy and privacy requirements elicitation?
2. RQ.2. What is the perception of privacy among ICT practitioners involved in software development projects?
3. RQ.3. How do ICT practitioners interpret and implement the concept of privacy in their daily activities? (That is, current privacy practices adopted by the organization.)
4. RQ.4. How do ICT practitioners interpret the concept of privacy in the light of the LGPD to be implemented in 2020? (That is, future privacy practices.)

### 3.3. Systematic Literature Review

To answer the first research question (RQ.1), we performed a Systematic Literature Review (SLR) in order to identify the methodologies and techniques used in the literature to software privacy and privacy requirements and for identifying the proposed privacy requirements’ elicitation methodologies. SLR was conducted according to the guidelines proposed by Kitchenham [32,33]. Primary studies are the ones that contribute to a systematic review. On the other hand, a systematic review is a form of secondary study [34]. During the SLR, the Planning, Conducting, and Reporting phases of the results were followed [34]. We used the tool StArt (State of the Art through Systematic Review, http://lapes.dc.ufscar.br/tools/start_tool) to boost the performance of the SLR, helping in the preparation and conduction steps of the Systematic Literature Review.

#### 3.3.1. Search Strategy

The SLR was performed in order to identify what methodologies are proposed in the literature regarding software privacy and privacy requirements elicitation. The search approach required Automatic Search [35], which consists of using a Search String to search across electronic databases, followed by a Manual Search [35]. Consequently, we implemented a manual exploration in journals and conferences/workshops. We defined the subsequent search string with proper search terms, in the automatic search: ((“privacy requirements” OR “data privacy” OR “software privacy”) AND (“software development” OR “Compliance” OR “LGPD” OR “GDPR”)). In particular, the Automatic Search was performed in the following databases: Digital library ACM (http://dl.acm.org/); Digital Library IEEE Xplore (http://ieeexplore.ieee.org/Xplore/home.jsp); DBLP-Computer Science Bibliography (http://dblp.uni-trier.de/); Scopus (https://www.scopus.com/home.uri).

#### 3.3.2. Selection Criteria

To identify relevant primary studies in the current software privacy and privacy requirements’ methodologies research, we defined the following inclusion and exclusion criteria for the primary studies selection: (1) The availability of the paper in the previously defined digital databases; (2) Studies published between 2009 and 2019. However, we also considered classical literature with definitions (books with classical concepts or pioneering articles); (3) The language of the study must be English or Portuguese; (4) The study should propose or evaluate methods, technologies, or privacy requirements’ methodologies. Moreover, we considered, as exclusion criteria of the studies, papers published as Short Paper that do not present sufficient information to extract the expected data, thus impairing the quality or relevance of the work.

#### 3.3.3. Screening Process

The SLR’s goal is to collect the relevant papers which adequately address the research questions, also known as primary studies. In this research, we performed both automatic and manual procedures in order to remove repeated entries. Then, the involved research read the paper titles, abstracts, and keywords to apply the inclusion criteria. Finally, we applied the exclusion criteria after thoroughly reading the papers and generated the list with primary studies.

#### 3.3.4. Data Extraction

When performing the data extraction, the involved researchers carefully read the primary studies. Moreover, we put the peer-review process in place, and two researchers elicited data for the same study. A third researcher resolved disagreements. We performed a pilot of the data extraction, aiming to align the understanding of the researchers for answering the research questions. We conducted the pilot with six randomly chosen primary studies, and the researchers examined the disagreements on the individual answers.

A total of 70 primary studies were identified. Given the collection of primary studies, the researchers fully read the studies and extracted the essential data. First, we generated a list of privacy requirements’ methodologies that were evidenced by each paper as presented in Table 3. We related each methodology for privacy requirements to information concerning the study and the type of evidence that we used to indicate it. After this association, we linked each privacy requirements methodology to text portions that supported it in the papers in which they were identified. After a complete reading of all selected articles in SLR, a total of **36 primary studies** were selected to be used in data extraction, as shown in Table 2.

In order to answer questions RQ.2, RQ.3, and RQ.4, we held an online survey with practitioners of ICT from some organizations in which we focused our study. We asked 27 closed-ended questions mainly using a Likert scale [55] and 03 open-ended questions. We made the survey available for one month, approximately. All participants allowed us to use and disclose the provided information while conducting the research, and participation was voluntary. The expected time to complete the survey was from 10 to 12 min. Sixty-eight ICT practitioners answered all the questions from our questionnaire.

## 4. Results and Discussion

In this section, we present the foremost findings of our study by answering the general research questions (RQ) that we investigate.

### 4.1. RQ.1. According to the Literature, What Are the Methodologies and Techniques Used to Conduct Software Privacy and Privacy Requirements’ Elicitation?

A number of methodologies that deal particularly with privacy issues have been introduced to support privacy requirements elicitation or software privacy, from the early stages of software design. Table 3 presents the methodologies proposed in the literature to conduct privacy requirements’ elicitation and software privacy.

Salnitri et al. [50] presented a software tool that facilitates the elicitation of privacy and security requirements, providing standards to identify common problems in software applications. The tool developed by the authors provides a method to enforce privacy and security requirements. Peixoto et al. [51] investigated the personal factors that affect the understanding of the privacy requirements by software developers. The authors conducted thirteen interviews at six private companies and found nine personal factors that affect how software developers perceive and interpret privacy requirements. Alkubaisy et al. [52] proposed a framework to identify and resolve conflicts between security and privacy requirements. The authors use patterns to describe the problem and propose a solution to each conflict encountered by the framework.

We have also found some works in the literature that report on the use of use cases, business process modeling, class diagrams, user story, user experience, design thinking, among others, for software privacy and privacy requirements’ elicitation [44,48,49,56,57,58] and have related works to software privacy and privacy requirements for cloud deployment models [46,47,59] and Internet of Things (IoT) [41,42,60,61]. This diversity of works found in several areas [43,45,62] demonstrates how important the elicitation of software privacy and privacy requirements is during the software development process and the growing concern of researchers from various fields of knowledge regarding the privacy of user data.

**Figure 1:**
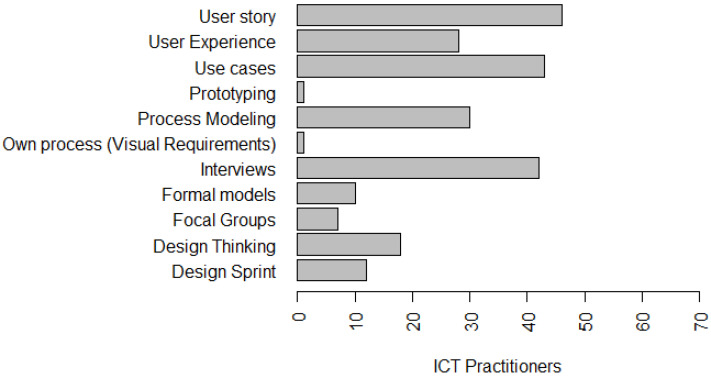
Techniques and Tools used to elicit and document requirements.

In order to ascertain what techniques are also being used in the industry to conduct software privacy and privacy requirements elicitation, in our survey, we elaborated a question to investigate what techniques and tools are currently used by software developers when conducting their activities to elicit and document software requirements. Figure 1 presents the results obtained. In Figure 1, it is possible to identify that 69.7% of development teams use User story to perform specification and documentation of requirements. In addition, 65.2% use Use Cases, 63.6% of teams use Interviews, 45.5% use Process Modeling, 42.4% use User Experience, 27.3% use Design Thinking and 18.2% use Design Sprint, 16.4% use Formal models, 11.9% use Focal groups, and 1.5% use prototyping and own process.

### 4.2. Survey Results

**Figure 2:**
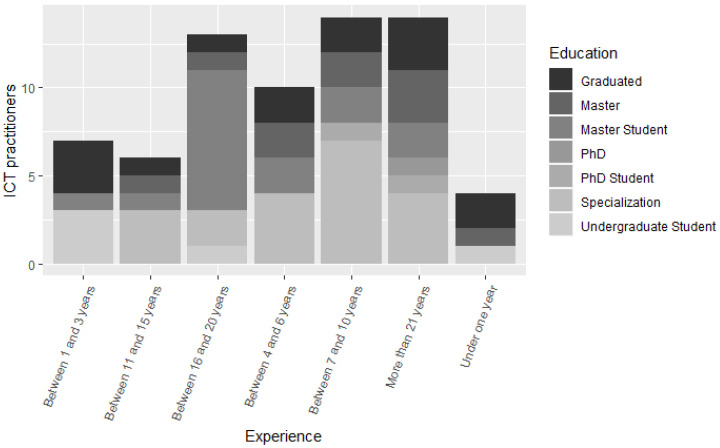
Respondents’ profile.

Questions from RQ.2 to RQ.4 were answered from the replies of the ICT practitioners obtained from the survey. In total, 68 professionals answered the survey. In addition, 13.2% of participants are between 21 and 25 years old, 22.1% are between 26 and 30 years old, 14.7% are between 31 and 36 years old, 25% are between 37 and 42 years old, 8.8% are between 43 and 47 years old, 7.4% are between 48 and 54 years old, and 8.8% are between 55 and 60 years old. Furthermore, 7.4% of participants are Undergraduate students, 20.6% are Graduates, 29.4% have a Specialization course, 23.5% are Masters students, 14.7% are Masters, 2.9% are PhD students, and 1.5% of respondents are doctors. In addition, 5.9% of participants have less than one year of experience, 10.3% between 1 and 3 years, 14.7% between 4 and 6 years of experience, 20.6% between 7 and 10 years, 8.8% between 11 and 15 years, 19.1% between 16 and 20 years, and 20.6% have over 21 years of experience. According to the results obtained in the survey, it is possible to state that the profile of the ICT practitioners who answered the survey is of highly experienced professionals, with 85% having more than four years of experience in the software development area, as shown in Figure 2.

Furthermore, we identified respondents’ profiles regarding the roles they have or had in systems development, such as Requirements Analyst, Project Manager, Software Engineer, Programmer/Developer, Tester, Designer (Interaction Designer, UX designer), Specialist in Human–Computer Interaction, among others. Only 4.4% of respondents had never been part of any software development projects. In addition, 38.09% have performed a single role so far. However, 28.57% performed from 2 to 3 roles, 12.69 % performed 4 roles, and 19.04 % from 5 to 8 different roles in the software development projects in which they participated. The most commonly performed roles are: 60.3% programmer/developer, Requirements Analyst (35.3%), Project Manager (33.8%), Software Engineer (32.4%), Data Modeler (30.9%), and Tester with 27.9%.

*Summary: The results obtained show that most ICT practitioners participating in the survey have, in addition to their academic background and years of experience in ICT, practical knowledge in several roles related to software development.*

**Figure 3:**
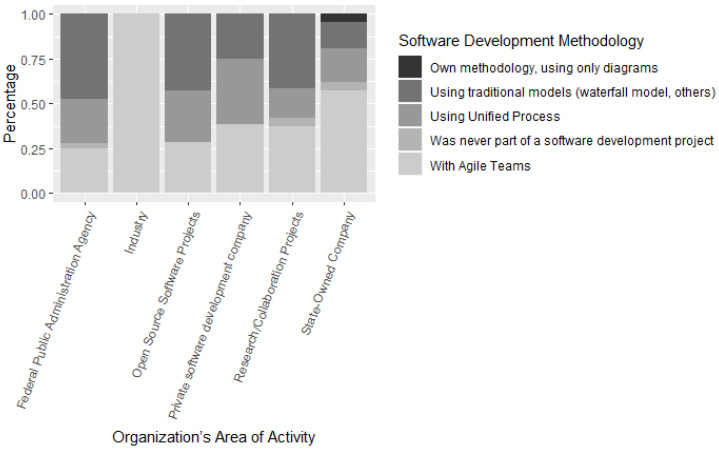
Organization’s area of activity and software development methodology.

Regarding the software development methodology used by the software development teams interviewed, 73.5% of ICT practitioners participated in projects with agile teams, 51.5% participated using the Unified Process and 64.7% of teams were part of projects using traditional models. (Waterfall model, etc.) as shown in Figure 3.

*Summary: This result allows us to conclude that most development teams currently work on projects that use agile methodology.*

Figure 3 presents the area of activity of the software development organizations in which the ICT practitioners participated in the survey act. As shown in Figure 3, 31.3% of participants work in State-Owned Companies, 37.3% work in Research/Collaboration projects with educational institution, 65.7% of participants work in private software development companies, 9% are from Open Source Software Projects, and 53.7% work in Federal Public Administration Agency (FPA). Most of the ICT practitioners interviewed work at a private software development companies or at Agencies of FPA.

#### 4.2.1. RQ.2. What Is the Perception of Privacy among ICT Practitioners Involved in Software Development Projects?

**Figure 4:**
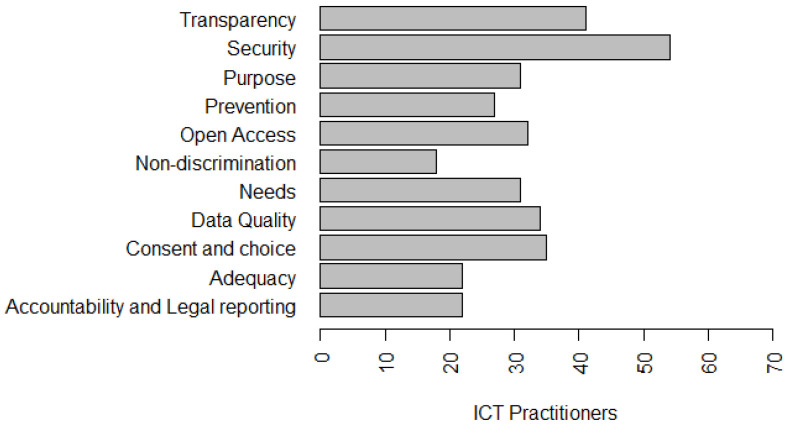
Privacy principles known to ICT practitioners.

To answer this question, we added some questions in the survey to gain a better understanding of the perception of ICT practitioners. Figure 4 presents the privacy principles that ICT practitioners are aware of. These principles are part of the LGPD and other privacy models. In addition, 62.1% stated that they knew about the principle of **Transparency**—guarantee data subjects of clear, accurate, and easily accessible information about the processing and their processing agents, in compliance with the trade and industrial secrets. In addition, 81.8% said they already knew the principle of **Security**—the use of technical and administrative measures to protect personal data from unauthorized access and accidental or illegal circumstances of destruction, loss, alteration, communication, or dissemination (Figure 4).

Forty-seven percent claimed to know **Purpose**—to perform data processing for legitimate, specific, explicit and informed purposes to the data subject, with no possibility of further processing in a manner incompatible with those purposes. In addition, 40.9% said they knew the principle of **Prevention**—taking measures to prevent the occurrence of damage due to the processing of personal data. Furthermore, 48.5% said they knew the principle of **Open Access**—guarantee data subjects free and easy information about the form and duration of processing, as well as the completeness of their personal data. In addition, 27.3% stated that they knew the principle of **Non-discrimination**—impossibility of carrying out processing for illegal or abusive discriminatory purposes (Figure 4).

Forty-seven percent said they have knowledge of the principle of **Needs**—limiting the processing of data to the minimum necessary to achieve their purposes, proportionate and not excessive. In addition, 51.5% said they knew the principle of **Data Quality**—guarantee data subjects of accuracy, clarity, relevance, and updating of data, according to the need and to fulfill the purpose of their processing. Fifty-three percent of participants said they knew the **Consent and Choice**—free, informed and unambiguous expression by which data subjects agree to the processing of their personal data for a specific purpose (Figure 4).

In addition, 33.3% said they knew the principle of **Adequacy**—compatibility of processing with the purposes informed to the data subject, according to the context of the processing and 33.3% stated that they were aware of the principle of **Accountability and Legal Reporting**—demonstration by the agent of the adoption of effective measures capable of verifying compliance with personal data protection rules, including the effectiveness of these measures (Figure 4).

Authors such as [14], identified **Security** as the most important principle in the perception of developers [3], ratifying the finding of this research (81.8%—Security). Moreover, knowledge of most of the principles related to LGPD by ICT practitioners points to a different result to that presented by Bednar et al. [13]. In such study, the authors identified, interviewing four senior engineers and two academic software team leaders, a low motivation of engineers to deal with privacy, study participants considered that privacy is a responsibility of the “legal world”, a concept very vague and a subject they have no interest in. Spiekermann et al. [63] also found that one out of three industry engineers shares a lack of enthusiasm for working in privacy.

**Figure 5:**
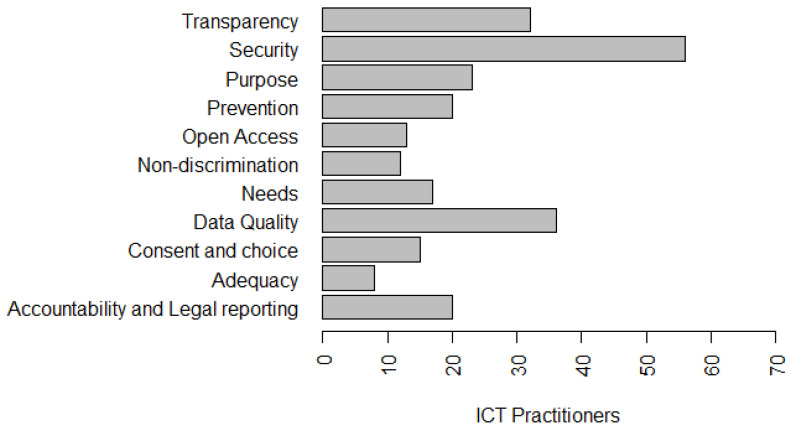
Privacy principles that ICT practitioners have worked with.

*Summary: In our study, awareness of more than 50% of privacy principles (six principles) that have been identified by more than 45% of ICT practitioners allows us to infer that, although LGPD is a recent law, it was created in 2018 and scheduled for implementation in August 2020—ICT practitioners are interested and motivated by the subject. It is worth highlighting that, in the survey, there is another question, similar to this one, but with a focus on ”LGPD principles being used in organizations“ (Figure 5). As indicated, the values are different, which allows us to infer that the knowledge of ICT practitioners on this subject does not only come from the use of privacy principles in the organizational environment.*

On questions related to the organizational environment and privacy practices, 48.5% strongly agree and 38.2% agree that the organizational environment interferes with privacy practices, while 13.2% were neutral. There was no disagreement regarding this issue, as shown in Q14 present in Figure 8.

In addition, 55.9% strongly agree and 36.8% agree that organizational privacy characteristics and procedures should be common knowledge to members of the organization, including software development teams. Only 7.4% of participants chose neutral, as shown in Q17 present in Figure 8.

Furthermore, ICT practitioners consider that it is important to disclose privacy solutions based on organizational policies, 33.8% strongly agree and 45.6% agree. Only 12% were neutral and 8.8% of participants disagree on this question, as shown in Q18 in Figure 8.

These answers ratify research conducted by Hadar et al. [3] and Ayalon et al. [14] that identified the organizational environment as a substantial factor, capable of influencing the behavior of software developers regarding privacy.

**Figure 6:**
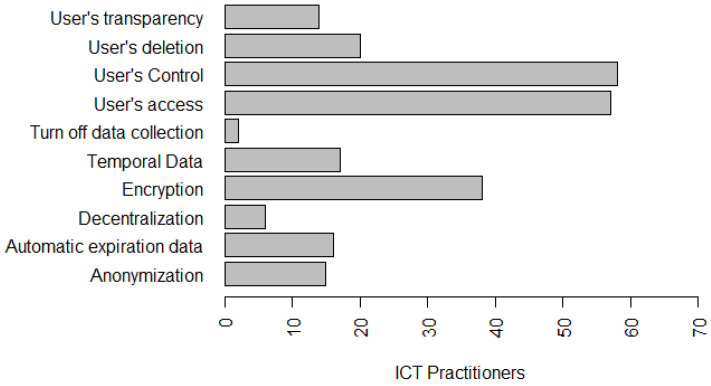
Privacy solutions that ICT practitioners have worked with.

With respect to the perception of the understanding of privacy, privacy requirements and issues related to security, 33.8% strongly agree and 45.6% agree that they have a partial understanding of the subject. Figure 4, Figure 5 and Figure 6 present the privacy principles and solutions best known to survey participants’ ICT practitioners. This finding allows us to conclude that organizations need to invest in the training and qualification of their professionals who work in the software development process, since, according to data privacy laws, all organizations need to develop their products in compliance with the legislation. This investment in qualification can be made through training and specific projects to implement organizational policies, such as data privacy policies, and by customizing the seven principles of the Privacy by Design (PbD) [64]:

1. proactive not reactive, which focuses on prevention;
2. privacy as the default setting;
3. privacy embedded into design;
4. full functionality;
5. end-to-end security;
6. visibility and transparency;
7. respect for user privacy.

Cavoukian [64] says in his study that several organizations have transformed these seven principles into specifications, recommendations, and practical suggestions of applications to use in different projects. Following these principles, some organizations use the practice of conducting continuous training of their practitioners to encourage the long-term involvement of their practitioners with the organizational data privacy policy. Amorim et al. [65] suggest the use of gamification techniques as an alternative to provide data privacy training to practitioners of an organization. Mavroeidi et al. [53] also investigated the use of Gamification for privacy requirements elicitation and the engagement of the users.

Perera et al. [54] proposed, using the Privacy-by-Design framework, a guide with a set of best practices to help software engineers to ensure data privacy of the users during the development of IoT applications. The guide was applied in different scenarios, demonstrating that the suggestions comply with the data privacy laws of several countries, including GDPR. In addition, the guide helps software engineers to manage the complexity of IoT application designs. The guide also provides guidelines for dividing the workload among software engineers (e.g., each engineer may focus/specialize in addressing a few guidelines). The guide can be used as a joint knowledge base to discuss IoT projects between teams.

*Summary: Most ICT practitioners believe they have a partial understanding of privacy. Most ICT practitioners identify that the organizational environment interferes with privacy practices and that the definition and disclosure of organizational privacy policies for practitioners are essential. Privacy principles with over than 45% of knowledge from ICT practitioners include Consent and Choice, Purpose, Need, Security, Data Quality, Transparency, and Open Access. Privacy solutions include User Control, User Access, and Encryption with more than 50% of understanding from ICT practitioners.*

#### 4.2.2. RQ.3. How Do ICT Practitioners Interpret and Implement the Concept of Privacy in Their Daily Activities? (That Is, Current Privacy Practices Adopted by the Organization)

In addition, 79.4% of ICT practitioners work or have worked on software feature development that had concerns related to data privacy and only 20.6% said they had never worked on data privacy issues.

Regarding the principles of data privacy that are being used in organizations in which ICT practitioners work, 23.8% stated that it was the Consent and choice principle; 36.5% stated it to be the principle of Purpose; 27% said it was the principle of Need; 88.9% said it was the principle of Security; 57.1% stated it to be the principle of Data quality; 50.8% said it was the principle of Transparency; 20.6% said it was the principle of Open access; 31.7% stated it to be the principle of Accountability and Legal reporting; 31.7% claimed it to be the principle of Prevention, 19% claimed it to be the principle of Non-discrimination, and 12.7% claimed it to be the principle of Adequacy, as presented in Figure 5.

Hadar et al. [3] identified the importance developers have with respect to security in their research, and infer that the use in practice of existing technology strategies for preserving privacy can influence and be influenced by developer perceptions. According to these authors, the perception of the strong association of privacy and security may influence developers to use better known solutions to solve privacy problems, namely, adopting security-related solutions may be a tendency to address any privacy concerns.

Figure 6 presents the privacy solutions that ICT practitioners have worked or are currently working with in the organizations in which they work—56.7% work with Encryption, 20.9% work with User’s Transparency, 86.6% work with User’s Control, 85.1% work with User’s Access, 3% work with Turn off Data Collection, 23.9% work with Automatic Data Expiration, 22.4% work with Data Anonymization, 9% work with Decentralization, 29.9% work with User’s Deletion, and 25.4% work with Temporal Data.

Ayalon et al. [14] and Hadar et al. [3] had already identified in their studies that encryption, user’s Control and user’s Access solutions as the most widely used and well-known practices, in the perception of the developers. This ratifies part of our results. This allows us to infer that organizations are slowly implementing the privacy principles/solutions related to LGPD, although their implementation date is close—August 2020. The most implemented principles, by practitioners, have been used for several years by organizations, security-related ICT, and also mentioned by the authors Ayalon et al. [14] and Hadar et al. [3]. One of the reasons for this delay might be related to the method of understanding the requirements collected from juridical texts because it is a complicated and error-prone process [66], and, as the legal requirements are often too abstract, they may leave space for multiple interpretations. Considering this, we ratify that it is crucial for effectively implementing privacy that developers should know laws, regulations, and organizational privacy policies [13,14]. We believe that organizational and managerial knowledge also seems to play an important role in the effective implementation of privacy policies. Figure 1 presents the tools used by software development teams to elicit and document requirements, as detailed in Section 4.1.

*Summary: Most developers have already worked with privacy solutions. Regarding the use of LGPD principles, ICT practitioners have identified as the most commonly used LGPD principles for organizations: Security; Data Quality; and Transparency. In addition, among the privacy solutions most commonly used by ICT practitioners are: User’s Control, User’s Access, and Encryption over 56%.*

#### 4.2.3. RQ.4. How Do ICT practitioners Interpret the Concept of Privacy in the Light of the LGPD to Be Implemented in 2020? (That is, Future Privacy Practices)

**Figure 7:**
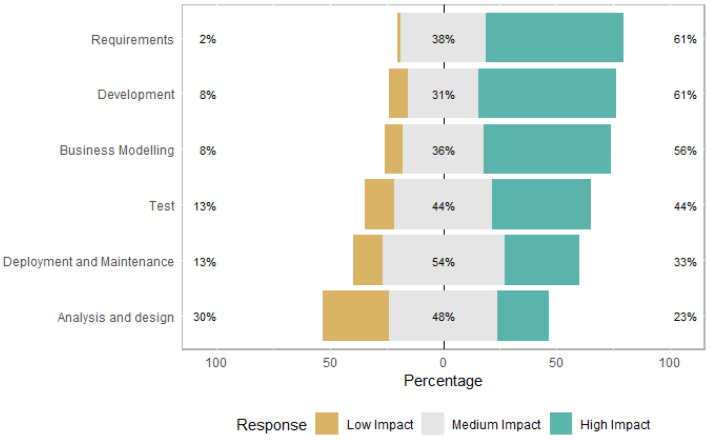
Impact on software development disciplines.

Figure 7 presents the perception of ICT practitioners regarding the impact on software development disciplines due to the changes proposed by LGPD. According to ICT practitioners, the disciplines that will be most impacted are the Requirements and Development disciplines, followed by the discipline of Business Modeling, Test, Deployment and Maintenance, and Analysis and Design. Interestingly, the Deployment and Maintenance discipline received the highest medium impact rating compared to the changes proposed by LGPD. Hadar et al. [3] identify, using the literature, some areas that would suffer the most impact, which were test, defects, and maintenance. Our research goes further by identifying the perceptions of those involved in the software development process about the impact levels and in which disciplines they will occur.

**Figure 8:**
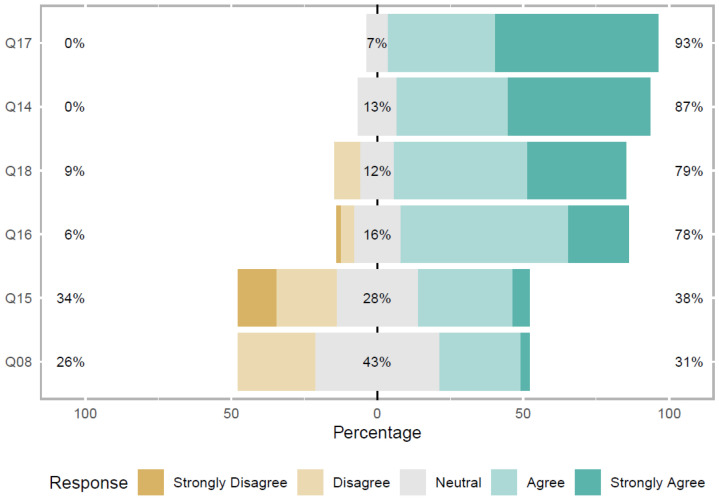
Perception of ICT practitioners.

In addition, 2.9% of ICT practitioners strongly agree and 27.9% agree, 43% were Neutral and 26.5% disagree that their understanding of the General Data Protection Act (LGPD), to be implemented in 2020, is sufficient for developing their activities in the software projects in which they work, as shown in Q8 present in Figure 8. This result is in line with research by Bednar et al. [13] which identified developers’ difficulty in understanding and following new privacy-related regulations. Notice that the research developed by Bednar et al. [13] was carried out with practitioners from organizations in various European countries (Germany, Italy, England, etc.), and with practitioners from the United States of America. The authors focused their study on the GDPR, which is similar to the LGPD, as we mentioned in Section 2.4. Data protection laws around the world have several privacy principles that are common to all countries. Considering that LGPD is the most recent legislation, it is possible to infer that this knowledge, with the implementation of the law in 2020, will tend to increase, despite the research carried out by Bednar et al. [13] still identifying the existence of difficulties related to the regulation of the privacy principles of user data, determined by GDPR, which has already been implemented for approximately two years (May 2018).

It is important to highlight the profile of the survey respondents, regarding their academic background, years of professional experience, and the roles performed. Most ICT practitioners have practical knowledge in various functions related to software development; 59% of participants performed from 2 to 8 roles (Developer, Requirements Analyst, Project Manager, Software Engineer, Tester) in software development projects. This finding allows us to infer a greater credibility from the results obtained, including those related to the impact on software development disciplines with regard to privacy and LGPD, since most participants in this research have academic background and practical experience in ICT.

Furthermore, 5.9% of ICT practitioners strongly agree and 32.4% agree, 13.2% strongly disagree, 20.6% disagree and 28% were neutral that the organization in which they work has informed team members about the LGPD and its implementation in 2020 and discussed the possible changes needed in their current and future systems, as shown in Q15 present in Figure 8. The answer to this question allows us to infer that the great diversity of answers must be related to the variety of organizations (where respondents work) that participated in the survey. One of the possible reasons for this result is that most participants are ICT practitioners from FPA agencies and state-owned companies, m part of the Brazilian Federal Public Administration and are likely to know the law because of government disclosure. This result allows us to conclude that organizations need to disclose guidelines and good practices to implement data privacy, making practitioners in the software development community aware of the importance of developing products complying with legislation. In addition, organizations must provide their professionals with specific training related to data privacy laws.

In addition, 20.6% of ICT practitioners strongly agree and 57.4% agree, 1.5% strongly disagree, 4.4% disagree, and 16.2% were neutral that LGPD should change the way teams work, as it will be required to implement several features and controls regarding consent, documentation, privacy accountability, as shown in Q16 present in Figure 8. These results confirm the findings reported by Dennedy et al. [17].

*Summary: Regarding the impact on software development disciplines due to changes proposed by LGPD, the ones identified as of greater impact were: the Requirements and Development, Business Modeling, Test, Deployment and Maintenance, and Analysis and Design disciplines. For most ICT practitioners, LGPD must change the way that development teams work. Some questions were not agreed upon, namely: whether ICT practitioners’ knowledge of the LGPD is sufficient for the development of their activities on software projects and whether the organization has informed practitioners about the LGPD.*

## 5. Threats to Validity

We cannot guarantee that all relevant primary studies were selected while conducting the SLR. It is possible that some relevant studies have not been selected. To mitigate this threat, we perform an automatic database search using the search string and complement the search for work in the area by manual searching, in order to attempt to collect all primary studies related to privacy requirements or methodologies used to software privacy and privacy requirements’ elicitation.

Another threat to validity of the paper is the possibility that the authors have introduced their bias in the data collection process. In this sense, the process of analysis of the collected data was performed jointly by all researchers. All researchers reviewed and analyzed intermediate results (primary studies and survey results). This iterative process was repeated until the end of the data collection and analysis process. We also held some meetings to validate the findings.

Regarding the survey, conclusions obtained derived from a limited number of ICT professionals working in the area of software development in some organizations located in Brasília, Federal District. At first, this seems to pose a major threat when trying to generalize the population, as our population is comprised of ICT practitioners with extensive knowledge and experience in software development, working in public, private organizations, or both. We believe that this threat has been mitigated. However, we are also aware that the survey could have been conducted in different organizations and locations with a population of inference consisting of professionals from various fields of knowledge.

## 6. Conclusions

In this paper, we conducted a systematic literature review to identify the methodologies proposed in the literature to software privacy and privacy requirements’ elicitation. We have found works in the literature that propose some methodologies and/or the use of techniques for software privacy and privacy requirements elicitation. We did not find industry reports using the methodologies and techniques found in the literature, as well as studies reporting the benefits of their practical application.

We also conducted a survey to understand the perception of ICT practitioners working in the software development area regarding the Brazilian General Law of Personal Data Protection, software privacy, and privacy requirements, and how the organizations in which they work are handling the need to develop LGPD-compliant systems. Most ICT practitioners who responded to the survey have more than four years of experience and 59.4% work as software developers. Survey results show that most ICT practitioners are aware that LGPD should change the way software development teams work and that these changes will have a high impact on the disciplines of the software development process, especially in the requirements’ elicitation and development discipline. These disciplines are directly related to the understanding of software privacy and privacy requirements.

ICT practitioners have stated that they lack the knowledge necessary to implement privacy principles and LGPD guidelines. Furthermore, organizational environment in which they work interferes with privacy practices. In addition, most ICT practitioners report that they use Use cases, User stories, and interviews to elicit and document software privacy and privacy requirements.

Findings of this work also demonstrate the need for software development organizations to inform their members about LGPD, as organizational privacy characteristics and procedures should be known to all, including software development teams. Thus, we can conclude that organizations need to disclose their privacy solutions based on their organizational policies. In addition, organizations must provide their professionals with specific training related to data privacy laws. Considering the results of other works related to GDPR found in the literature, in which several principles are similar to LGPD, we conclude that this is a global need for software development organizations, regardless of which privacy model is adopted—GDPR or LGPD. Concerning this conclusion, we are aware that more studies are needed to ratify or rectify if the principles related to the software development process determined by LGPD and GDPR are similar and/or complement each other.

As future work, we intend to conduct a survey with a broader population and perform a controlled experiment in a software development organization. This organization has to meet the needs and guidelines imposed by LGPD and GDPR during software development, by gathering insights of the team members and recording the challenges encountered during the privacy requirements’ elicitation.

## Figures and Tables

**Table 1:** Comparison between the principles and bases of the LGPD model [18] and GDPR [19,31].

LGPD	GDPR
**Purpose**: execution of the processing for legitimate, specific, explicit and informed purposes to the data subject, with no possibility of further processing in a manner incompatible with those purposes.	**Purpose limitation**—“collected for specified, explicit and legitimate purposes and not further processed in a manner that is incompatible with those purposes; further processing for archiving purposes in the public interest, scientific or historical research purposes or statistical purposes shall not be considered to be incompatible with the initial purposes”.
**Adequacy**: Agreement of processing with the purposes reported to the data holder, being consistent with the context of the processing.	**Storage limitation**—“kept in a form which permits identification of data subjects for no longer than is necessary for the purposes for which the personal data are processed; personal data may be stored for longer periods insofar as the personal data will be processed solely for archiving purposes in the public interest, scientific or historical research purposes or statistical purposes subject to implementation of the appropriate technical and organizational measures required by the GDPR in order to safeguard the rights and freedoms of individuals”.
**Needs**: Limitation of the treatment to the minimum necessary to achieve its ends, with coverage of relevant data, proportional and not excessive concerning the data processing purposes;	**Data Minimization**—“adequate, relevant and limited to what is necessary in relation to the purposes for which they are processed”.
**Open Access**: Assurance to data subjects, free and accessible information about the form and duration of data processing, as well as the completeness of their sensitive data.	*Individuals have the right to access their personal data. *Not considered a principle, but a right.
**Data Quality**: Ensure data holders the accuracy, clarity, relevance, and updating of the data, as necessary, and the purposes of its processing.	**Accuracy** (Accurate, up to date, erased or rectified)—“accurate and, where necessary, kept up to date; every reasonable step must be taken to ensure that personal data that are inaccurate, having regard to the purposes for which they are processed, are erased or rectified without delay”.
**Transparency**: Guarantee, to the data holders, of precise, reliable and readily available information on the execution of the processing and its corresponding processing agents, subject to commercial and industrial secrets.	**Lawfulness, fairness, and transparency**—“Personal data shall be processed lawfully, fairly and in a transparent manner in relation to the data subject “.
**Security**: Use of administrative and technical standards to protect personal data from unauthorized access and unexpected or unlawful situations of destruction, loss, alteration, communication, or dissemination.	**Integrity and confidentiality**—“processed in a manner that ensures appropriate security of the personal data, including protection against unauthorized or unlawful processing and against accidental loss, destruction or damage, using appropriate technical or organizational measures”.
**Prevention**: Adoption of means to prevent the appearance of damages due to the personal data processing.	*Right to be informed *Not considered a principle, but a right.
**Non-discrimination**: Inability to perform data processing for illicit or abusive discriminatory purposes. The data subject has the right to request a review of the decision, and the supervisory authority may examine it to check discriminatory aspects in the automated processing of personal data.	*Not considered a principle, but a right.
**Accountability and Legal reporting**: The agent demonstrates the adoption of effective measures, capable of proving the observance and compliance with personal data protection rules, including the effectiveness of such measures.	**Accountability** the controller will be responsible and will be able to demonstrate compliance with the GDPR.

**Table 2:** Selected primary studies at SLR.

ID	Title	Reference
E1	A privacy threat analysis framework: supporting the elicitation and fulfillment of privacy requirements	[5]
E2	Adapting the square process for privacy requirements engineering	[36]
E3	Pris Tool: A Case Tool For Privacy-Oriented Requirements Engineering	[8]
E4	A framework for modeling privacy requirements in role engineering	[4]
E5	STRAP: a structured analysis framework for privacy	[10]
E6	Security and Privacy Requirements Analysis within a Social Setting	[9]
E7	PCM Tool: Privacy Requirements Specification in Agile Software Development	[37]
E8	Computer-Aided Privacy Requirements Elicitation Technique	[38]
E9	Recommender-based privacy requirements elicitation – EPICUREAN:an approach to simplify privacy settings in IoT applications with respect to the GDPR	[39]
E10	Engineering Privacy by Design: Are engineers ready to live up to the challenge?	[13]
E11	Privacy by designers: software developers’ privacy mindset	[3]
E12	Privacy by Design Leading Edge	[40]
E13	How Developers Make Design Decisions about Users’ Privacy: The Place of Professional Communities and Organizational Climate	[14]
E14	Privacy and Data Protection by Design – from policy to engineering	[22]
E15	The challenges of privacy by design	[21]
E16	Comparing Privacy Requirements Engineering Approaches	[26]
E17	Addressing privacy requirements in system design: the PriS method	[6]
E18	Incorporating privacy in the design of cloud-based systems: a conceptual meta-model	[1]
E19	The Grace Period Has Ended: An Approach to Operationalize GDPR Requirements	[31]
E20	Security and Privacy Requirements Analysis within a Social Setting	[9]
E21	Model Based Process to Support Security and Privacy Requirements Engineering	[7]
E22	A framework for modeling privacy requirements in role engineering	[4]
E23	Privacy and security requirements framework for the internet of things (IoT)	[41]
E24	A taxonomy of security and privacy requirements for the Internet of Things (IoT)	[42]
E25	Privacy Policy Specification Framework for Addressing End-Users’ Privacy Requirements	[43]
E26	Enabling Users to Specify Correct Privacy Requirements	[44]
E27	Compliance Checking of Open Source EHR Applications for HIPAA and ONC Security and Privacy Requirements	[45]
E28	Security and Privacy System Requirements for Adopting Cloud Computing in Healthcare Data Sharing Scenarios	[46]
E29	A Semi-Automatic Approach for Eliciting Cloud Security and Privacy Requirements	[47]
E30	Specifying privacy requirements with goal-oriented modeling languages	[48]
E31	Modeling Security and Privacy Requirements: a Use Case-Driven Approach	[49]
E32	Modelling the interplay of security, privacy and trust in sociotechnical systems: a computer-aided design approach	[50]
E33	On Understanding How Developers Perceive and Interpret Privacy Requirements Research Preview	[51]
E34	Towards Detecting and Mitigating Conflicts for Privacy and Security Requirements	[52]
E35	The Role of Gamification in Privacy Protection and User Engagement	[53]
E36	Designing privacy-aware internet of things applications	[54]

**Table 3:** Privacy requirements’ engineering methodologies.

Methodology	Description	Tool	Reference
LINDDUN	LINDDUN aims to support the elicitation and fulfillment of privacy requirements in software based systems through a privacy threat analysis framework.	Design of a data flow diagram (DFD) of the system	[5]
SQUARE for Privacy	SQUARE for privacy follows the same steps as the original SQUARE method in conjunction with the Privacy Requirements Elicitation Technique (PRET).	PRET tool	[36]
PriS	PriS is referred as a goal-oriented approach that considers privacy requirements as organizational goals that have to be achieved by the system.	Pris	[8]
Role-Based Access Control (RBAC)	RBAC framework is an agent-oriented framework that aims to model privacy requirements and to map user’s roles and permissions with a structured way.	Not supported	[4]
STRAP	STRAP is referred as a goal-oriented approach and it is based on a structured analysis of privacy vulnerabilities, as well as on an iterative process of four steps (Analysis, Refinement, Evaluation and Iteration) for the integration of preferences.	Not supported	[10]
Secure Tropos with PriS	A model-based process that takes into consideration security and privacy concepts in parallel at the early stages of system analysis and design.	Not supported	[7]
i* method	Agent-oriented method that focuses on system agents and their interdependencies and aims to analyze, model, and design the organization’s processes at the early stages of system design.	Organization Modelling Environment (OME)	[9]
Privacy Criteria Method	Privacy Criteria Method (PCM)—an approach designed to guide the specification of privacy requirements in agile software development.	PCM Tool	[37]
Pret	Computer-Aided Privacy Requirements Elicitation Technique that helps software developers elicit privacy requirements more efficiently in the early stages of software development.	PRET tool	[38]
EPICUREAN	EPICUREAN is a recommender-based privacy requirements elicitation approach. EPICUREAN uses modeling and data mining techniques to determine and recommend appropriate privacy settings to the user.	EPICUREAN Knowledge Model	[39]
